# Strongly Selective Polymer Membranes Modified with Heteroarm Stars for the Ethylene Glycol Dehydration by Pervaporation

**DOI:** 10.3390/membranes10050086

**Published:** 2020-04-29

**Authors:** Valeriia Rostovtseva, Alexandra Pulyalina, Daria Rudakova, Ludmila Vinogradova, Galina Polotskaya

**Affiliations:** 1 Institute of Chemistry, Saint Petersburg State University, Universitetskiy pr. 26, Saint Petersburg 198504, Russia; st017536@student.spbu.ru (V.R.); rudakova.gda@gmail.com (D.R.); g_polotskaya@mail.ru (G.P.); 2 Institute of Macromolecular Compounds, Russian Academy of Sciences, Bolshoy pr. 31, Saint Petersburg 199004, Russia; vinogradovalv@rambler.ru

**Keywords:** pervaporation, ethylene glycol, poly(phenylene oxide), star-shaped macromolecules

## Abstract

Hybrid membranes based on poly (2,6-dimethyl-1,4-phenylene oxide) modified with heteroarm stars (HAS) were developed to separate ethylene glycol/water mixtures by pervaporation. The HAS consist of a small branching center fullerene C 60 and twelve arms of different nature, six arms of nonpolar polystyrene and six arms of polar poly-tert-butyl methacrylate. The changes of structure and physical properties with HAS inclusion were systematically studied using SEM, X-ray diffraction analysis, TGA, and contact angle measurements. Mass transfer of ethylene glycol and water through membranes was studied by sorption and pervaporation tests. It was found that the growth of HAS content up to 5 wt% in the membrane leads to an increase in the total flux and a strong increase in the separation factor. To evaluate intrinsic properties of the penetrant–membrane system, permeability and selectivity were calculated. Overall, utilizing star-shaped macromolecules as a filler can be a promising way to improve the separation performance of diffusion membranes.

## 1. Introduction

Ethylene glycol has been widely used in various economic and industrial fields, and great demand for it is constantly increasing. Among the major applications, it can be distinguished in the manufacture of polyethylene glycol, polyethylene terephthalate, polyester, cellophane, polyurethane [1], manufacture of non-volatile antifreeze, brake fluids, and anti-icing fluids. Annual world ethylene glycol (EG) production and consumption are about 30 million tons with an estimated increase of 5–10% per year.

The production of EG is commonly carried out by hydrolysis of ethylene oxide in the presence of a water excess [2]; the resulting product is 70 wt% EG aqueous solution, therefore, an additional stage of EG dehydration is required [3]. For several applications, especially in chemical processes, EG is required as a reagent of high purity. EG has a very high boiling point (197.3 °C), so the separation of EG/water mixture using traditional methods, such as multi-stage evaporation and distillation, is very complex and energy consuming [2,3,4]. The utilization of EG is complicated by its high toxicity and also due to problems of waste regeneration and disposal. Therefore, the search for an alternative method of EG dehydration and regeneration remains an urgent issue.

Pervaporation as a membrane-based separation method has attracted much attention due to its high efficiency, low energy consumption, and environmental friendliness [5,6,7,8]. Currently, researchers have highlighted the use of pervaporation for the purification of EG from water impurities, opening up great potential for highly pure EG production using membranes from poly(vinyl alcohol) (PVA)/zeolite 4A mixed matrix composite [9], chitosan/polysulfone composite membrane [10], dual-layer polybenzimidazole/polyetherimide membranes [11], membranes based on 1-butyl-3-methylimidazolium tetrafluoroborate (Bmim)(BF40 infiltrated into a buckypaper [12].

Application of hybrid materials for membrane fabrication improves operational performance, as well as mechanical and chemical stability, for instance, PVA-GPTMS/TEOS hybrid pervaporation membrane [13], PVA-silica nanocomposite membrane [14], NaA zeolite membranes [15], carbon nanotubes [16], polydopamine-coated metal-organic framework [17]. It has been shown that interfacial modification of inorganic fillers can be an effective way to increase both permeability and selectivity of membranes [17].

Among numerous membrane materials, poly(2,6-dimethyl-1,4-phenylene oxide) (PPO) has great potential for gas separation [18,19,20] and pervaporation [21,22] being an aromatic glassy polymer with high mechanical and thermal stability and good resistance to chemical agents. There are only a few known cases when PPO-based membranes had been applied for the dehydration of organic solvents [23,24,25]. PPO membranes modified with fullerene C_60_ (up to 2 wt%) are selective to water in the separation of ethanol/water and ethyl acetate/water binary mixtures [24,25].

The modernization of fullerene C_60_ by methods of anionic polymerization creates star-shaped macromolecules that are unique modifiers for membranes. The star-shaped macromolecule consists of a small fullerene C_60_ core and twelve polymer arms. Hybrid PPO membranes modified with star macromolecules composed of a C_60_ core and six polystyrene (PS) arms and six poly-2-vinylpyridine (P2VP) arms have provided enhanced selectivity in dehydration of acetic acid [26] and separation of the quaternary mixture (*n*-propanol/acetic acid/propyl acetate/water) [27]. It has been reported [27,28,29,30,31] that PPO membranes modified with star macromolecules exhibited enhanced pervaporation performance, presumably due to the presence of polymer arms with a different chemical nature (nonpolar and polar) in a modifier. The PS arms are fully compatible with the PPO matrix and a phase separation does not occur up to the temperatures of their thermal destruction [32]. Star modifiers significantly affect the internal structure and the free volume of the membranes [27].

In this study, heteroarm stars (HAS) containing six nonpolar arms of PS and six polar arms of poly-tert-butyl methacrylate (PTBMA) on a C_60_ core were selected as a modifier of the PPO matrix. The main aims of this work were the preparation and research of PPO/HAS hybrid membranes. The influence of a novel type of modifier on the transport properties of PPO-based membranes in the pervaporation of EG/water mixture was thoroughly discussed. Special emphasis was placed on studying membrane morphology and physicochemical parameters characterized by SEM, X-ray spectroscopy, TGA, and sorption studies.

## 2. Materials and Methods

### 2.1. Materials

Poly(2,6-dimethyl-1,4-phenylene oxide) (PPO) with a molecular weight 172,000 Da and density 1.06 g/cm^3^ (Polymer Institute, Brno, Czech Republic) was used. Chloroform and EG were obtained from Vecton (Saint Petersburg, Russia). All the chemicals were used without further purification.

**Figure 1:**
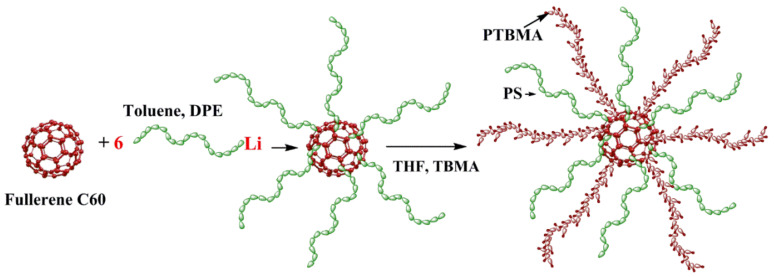
The synthesis of heteroarm stars (HAS), where DPE is 1,1-diphenylethylene, THF is tetrahydrofurane, and TBMA is tert-butylmethacrylate.

Heteroarm stars (HAS) (Figure 1) composed of a C_60_ core covalently bonded with six PS arms and six PTBMA arms were synthesized by methods of the anionic polymerization [33]. Individual PS arms were characterized by size exclusion chromatography (*M*_n_ = 6.9 × 10^3^ Da and *M*_w_/*M*_n_ = 1.04). The molecular weight of the PTBMA arm was equal to that of PS arm.

### 2.2. Membrane Preparation

For PPO/HAS membranes preparation, a solution-casting method was used. Firstly, solutions of PPO and HAS were prepared by dissolving in chloroform with a concentration of 3 wt%, and then sonicated for 30 min (ultrasonic bath Sapphire, Moscow, Russia). After that, these solutions were mixed in appropriate amounts to obtain the required content of the modifier in the membrane (2 and 5 wt%). The resulting solution was stirred for 2 h and left for 2–3 days to complete the interaction between PPO and HAS. Then, the mixture was stirred for 40 min and filtered. It was cast onto the cellophane support and the solvent was slowly evaporated at 40 °C. The membranes were peeled off from the cellophane and put in the vacuum oven at 40 °C to remove traces of the solvent for at least one week. The thickness of the membranes was about 40 μm. The residual solvent content in the film was 0.3 ± 0.1 wt%.

### 2.3. Membrane Characterization

Images of surface and cross-sections of membranes were observed by a Zeiss SUPRA 55VP scanning electron microscope (SEM) (Carl Zeiss, Oberkochen, Germany) equipped with In-lens SE and SE2 secondary electron detectors, a secondary electron detector for low vacuum mode (VPSE), and a four-quadrant backscattered electron detector (AsB). Before tests, samples were coated with a 20 nm thick carbon layer using the Quorum 150 cathode sputtering installation (Quorum Technologies Ltd., Lewes, UK).

Thermogravimetric analysis (TGA) of the PPO composite membranes was performed using a TG 209 F3 Iris thermo-microbalance (Netzsch, Selb, Germany) at a heating rate of 10 °C/min from room temperature to 800 °C in a nitrogen atmosphere.

X-ray diffraction (XRD) analysis was carried out on an X-ray diffractometer D8 DISCOVER (Bruker, Bremen, Germany) using CuKa radiation with a wavelength of 1.54 Å. Scans were made with a step size of 0.058 ranging from 5 to 50°.

The density measurements were performed using the flotation method with a laboratory-made unit and water/saccharose solutions. At least two samples of each membrane (0.05–0.10 g) and five measurements were used to calculate the average density with a minimal error.

The contact angles of water and EG on the membrane surfaces were measured via the sessile drop technique using a Drop Shape Analyzer DSA 10 (Krüss, Hamburg, Germany) at room temperature and atmospheric pressure.

### 2.4. Sorption Study

The sorption experiment was carried out by the immersion of the samples in a pure liquid at atmospheric pressure and room temperature 25 °C. Samples were removed from the liquid at regular intervals and blotted with a tissue paper, and then they were weighed on an analytical balance with an accuracy of 10^−4^ g. The experiment continued until the equilibrium state was achieved. 

The total amount of liquid sorbed was determined using the following equation:

(1) $$S=\frac{M_{S}-M_{D}}{M_{D}}\times 100\%$$

where *M_s_* is the weight of the swollen membrane and *M_d_* is the weight of the dry membrane.

The Hildebrand solubility parameters of the membrane polymers were calculated using the cohesion energy *E*_coh_, and the molar volume *V*_m_, of individual groups constituting the polymer molecules, as given by the following equation [34]:

(2) $$\delta_{i}={\left(\frac{\sum E_{coh,i}}{\sum V_{m,i}}\right)}^{\frac{1}{2}}$$

### 2.5. Pervaporation

Pervaporation experiments were conducted via a lab-scale apparatus with stirring. The effective area of the membrane supported by a metal disk with small gaps was approximately 14.8 cm^2^. Downstream pressure below 10^−2^ mm Hg was maintained on the permeate side with vacuum pump MD 1C (Vacuubrand GMBH, Wertheim, Germany). The ethylene glycol concentration in the feed ranged from 80 to 95 wt%. The permeate was collected into a trap immersed in liquid nitrogen and weighted with the balance Mettler Toledo ME204 (Mettler Toledo, Columbus, OH, USA). The feed and product concentrations were analyzed using a gas chromatograph Chromatec Crystal 5000.2 (Chromatec, Yoshkar-Ola, Russia) with a thermal conductivity detector. The experiments were repeated 3 times and the average value of the results was considered.

Total flux through the membrane was determined as follows:

(3) $$J=\frac{Q}{A\times t}$$

where *Q* is the amount of product (g), *A* is the effective membrane area (m^2^), and *t* is the operating time (h).

The separation factor *β_Water/EG_* was defined according to the equation [35]:

(4) $$\beta_{Water/EG}=\frac{Y_{Water}/Y_{EG}}{X_{Water}/X_{EG}}$$

where *Y* and *X* are the weight fraction of component in the permeate and feed, respectively.

Pervaporation separation index, *PSI*, was calculated as:

(5) $$PSI=J\cdot \left(\beta -1\right)$$

In addition, the membrane intrinsic properties can be evaluated in terms of the permeability, *P_i_*, and the membrane selectivity, *α*, which were calculated using the following equations [36]:

(6) $$P_{i}=j_{i}\frac{l}{p_{i0}-p_{i}}$$

(7) $$\alpha_{Water/EG}=\frac{P_{Water}}{P_{EG}}$$

where *p_i0_* and *p_i_* are the vapor pressures of component *i* in feed and permeate, respectively; *l* is the membrane thickness; and *j_i_* is the molar flux of component *i*.

## 3. Results

### 3.1. Membrane Characterization

PPO/HAS membranes were prepared by a solution-casting method. The HAS modifier consists of C_60_ core and six nonpolar arms of PS and six polar arms of PTBMA. The peculiarity of HAS macromolecules is their ability to form clusters. It has been shown [37] that mutual penetration of PS and PTBMA arms took place in solutions, and supramolecular structures containing up to 12 HAS macromolecules with a diameter of ~50 nm were generated. These clusters interact with the PPO matrix mainly due to the affinity of PS arms and PPO, which are completely intersoluble and the phase separation does not occur until the temperature of their thermal destruction [38]; this fact determines uniform distribution of HSM in PPO matrix.

The volume of HAS clusters and their interaction with PPO matrix affect the fine morphology and the free volume of the membrane. Therefore, when preparing PPO/HAS membranes from the solution in chloroform, the changes of membrane structure was expected as a result of polar arms segregation.

**Figure 2:**
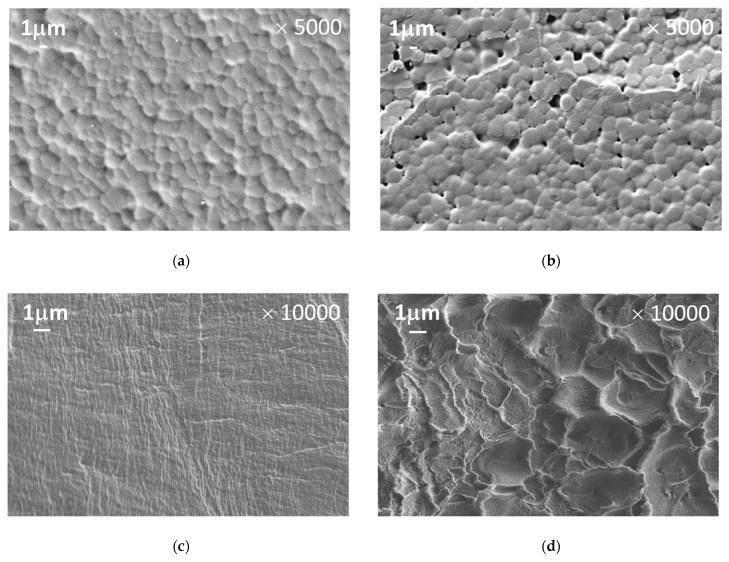
SEM images of (**a**,**b**) surface and (**c**,**d**) cross-section for (**a**,**c**) poly(2,6-dimethyl-1,4-phenylene oxide) (PPO) and (**b**,**d**) PPO/HAS (5%) membranes.

The effects of HAS inclusion on the structure of PPO-based membranes were studied by SEM and X-ray diffraction analysis. Figure 2 shows SEM microphotographs of the surface and cross-sections for PPO and PPO/HAS (5%) samples. The surface structure of the membranes does not noticeably change after HAS inclusion into the PPO matrix. In contrast, when comparing cross-sectional images (Figure 2c,d), a significant change in the membrane morphology is observed. The relatively homogeneous morphology of the PPO membrane (Figure 2c) transforms into a pronounced cellular structure after its modification (Figure 2d).

**Figure 3:**
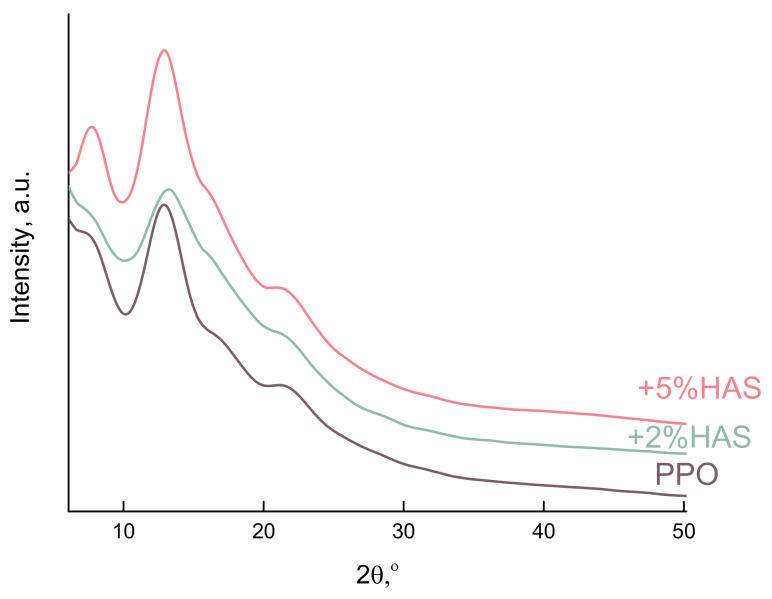
X-ray diffraction patterns of PPO/heteroarm stars (HAS) (0, 2, 5 wt%) membranes.

X-ray phase analysis was used to gain insight into the effect of the HAS modifier on membrane crystallinity. Figure 3 shows the XRD patterns of pristine PPO and modified PPO/HAS membranes. The broad peaks appearing at 2Θ~12° and 23° indicate a small content of the crystalline phase in the samples of our PPO-based membranes. A similar result on low crystallinity for PPO films prepared from a solution in chloroform was reported in [39]. For PPO/HAS (2 and 5 wt%) membranes, a slight shift of the peaks to the region of higher values of 2Θ is observed relative to the peak position of the PPO sample. But in general, the character of the XRD patterns does not change, which is explained by the preservation of the crystal lattice type during membrane modification.

**Figure 4:**
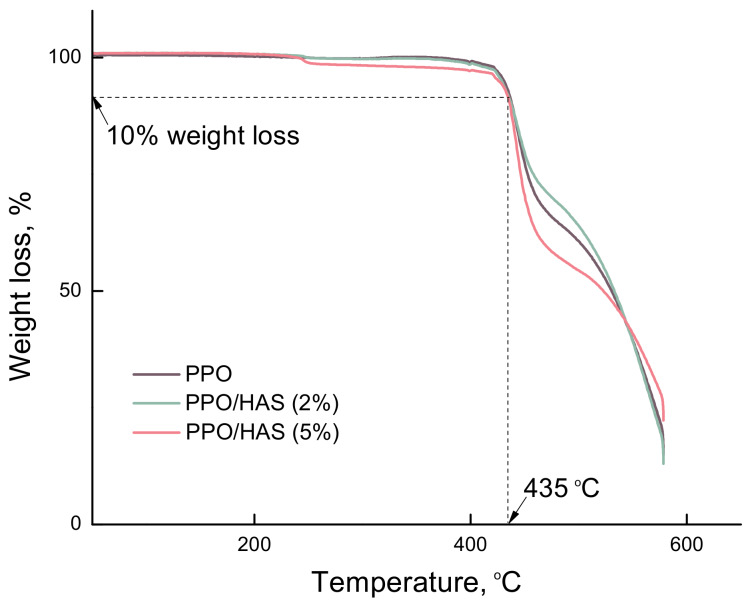
TG curves of PPO/HAS (0, 2, 5 wt%) membranes.

The thermal stability of the membranes was examined by the thermogravimetric analysis. Figure 4 shows the TG curves for PPO and PPO/HAS (2%, 5%) membranes during isothermal heating from 25 to 575 °C. All membranes showed almost the same tendency for the weight loss at heating. In the region up to 400 °C, a small weight loss ~4 wt% was recorded which is a result of the release of moisture and low molecular weight impurities sorbed on the surface of the membranes, as well as a result of the destruction of HAS arms. Thermal decomposition of the PS arms is possible at ~450 °C. PTBMA is another arm of HAS which is prone to depolymerization at heating, and destruction of PTBMA arms is observed at ~300 °C [40]. Some difference in the position of the destruction curves for PPO/HAS with different content of the modifier can be explained by the contribution of destructive processes of the star macromolecules. The highest weight loss up to 400 °C (~4 wt%) and at the region from 400 to 500 °C (up to 50 wt%) was recorded for PPO/HAS (5%).

Usually, 10% weight loss (τ_10_) serves as criterion for the thermal stability of the polymer samples; it is observed on the TG curves at ~435 °C for PPO/HAS samples. These data indicate the high thermal stability of PPO-based membranes in the temperature range at which pervaporation experiments are carried out. It is seen that the inclusion of a star-shaped modifier does not significantly affect the thermal properties of the membranes.

Several physical properties of membranes such as density, contact angles, and surface tension were determined and presented in Table 1. The density of the pristine PPO membrane is equal to 1.057 g/cm3 which is consistent with the literature data [29]. After modification, the density of the PPO/HAS membranes slightly increases. This trend in the density changing suggests good interaction between the HAS macromolecules and the PPO matrix.

Table 1 shows data on the contact angle of water and EG on the surface of the PPO and PPO/HAS (2%, 5%) membranes. Inclusion of a modifier and growth of the HAS content in the membrane increase values of the contact angles making the membrane more hydrophobic. The increase in hydrophobic quality of the surface is attributed to the nonpolar PS arms of the HAS. The values of critical surface tension for the membranes were calculated by the Owens–Wendt method using data on contact angles [41]. The polar contribution of surface tension σps, as well as dispersive contribution σds, decreases with the growth of HAS concentration up to 5 wt%. The total critical surface tension, σs, decreases for modified PPO/HAS membranes as compared with pristine PPO, which can be related to inhibiting the movement of polymer chains.

### 3.2. Transport Properties

The process of mass transfer through PPO and PPO/HAS (2%, 5%) membranes was studied relative to organic liquids of a different chemical nature, i.e., EG and water. Table 2 lists some physicochemical properties of these liquids. The problem of EG and water separation arises from the industrial process for its synthesis by hydrolysis of ethylene oxide in the presence of a water excess [2]; the resulting product is 70 wt% EG aqueous solution. The application of membrane technology of pervaporation is a more effective way to solve the problem of EG dehydration.

Parameters of EG and water mass transfer through the PPO-based membranes were determined in sorption and pervaporation experiments.

Sorption experiments were carried out by immersion of membrane samples in the individual liquids to determine the amount of liquid sorbed by the polymer. Table 3 lists data on the equilibrium sorption degree of EG and water in PPO and PPO/HAS (2, 5%) membranes. The sorption capability of all membranes in both water and EG increases with the growth of the HAS content in the membrane. The equilibrium sorption degree of EG in the membranes is approximately two times higher than that of water. This result is in agreement with the solubility theory and correlation of Hildebrand solubility parameters (*δ*) of liquids and membrane polymer.

Data on solubility parameters of EG and water are given in Table 2. The δ value of PPO is equal to 18.2 (J/cm^3^)^1/2^ [42], and δ values of HAS composing polymers, namely PS and PTBMA, are equal to 18.6 and 19.2 (J/cm^3^)^1/2^, respectively. According to the solubility theory [34], the smaller the difference in solubility parameters of polymer and liquid |Δδ|, the better the solubility of this liquid in the polymer. Therefore, the solubility of EG in PPO and PPO/HAS membranes is preferential as compared to water.

The main transport parameters of the PPO/HAS (0, 2, 5 wt%) membranes were determined in the pervaporation of EG/water mixtures at 50 °C. The influence of water concentration in the feed on the performance was investigated by carrying out experiments in the range of feed water concentrations from 5 to 12 wt% which serve the particular industrial interest, namely, the regeneration of ethylene glycol related to natural gas dehydration by ethylene glycol [4].

**Figure 5:**
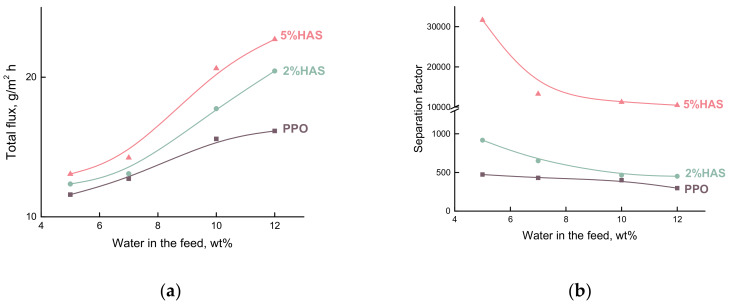
Dependences of (**a**) the total flux and (**b**) the separation factor (*β_water/EG_*) on water concentration in the feed for the pervaporation of ethylene glycol (EG)/water mixture using the PPO-based membranes, 50 °C.

Figure 5 shows the dependencies of the total flux and the separation factor on water concentration in the feed. It was found that all membranes are preferably permeable to water. According to the data in Figure 5a, the total flux increases with water concentration in the feed. The growth of HAS content in the PPO matrix leads to an increase in the total flux. It can be attributed to an altered packing of PPO chains by including aggregates of HAS macromolecules. Hence, a transformation of membrane structure and, in particular, the geometry of transport channels provides a facilitated transport of penetrant molecules through the polymer matrix of hybrid membranes.

As seen from Figure 5b, the separation factor (*β_water/EG_*) decreases with increasing water concentration in the feed. The inclusion of modifier up to 5 wt% HAS led to a more efficient separation of the EG/water mixture as compared with that of the PPO membrane.

It is taken to represent the transport properties of membranes in pervaporation not only in terms of total flux and separation factor but also in terms of permeability and selectivity [36]. The calculation of permeability and selectivity permits to normalize the membrane properties on driving forces and to reveal intrinsic properties of the studied penetrant-membrane system.

**Figure 6:**
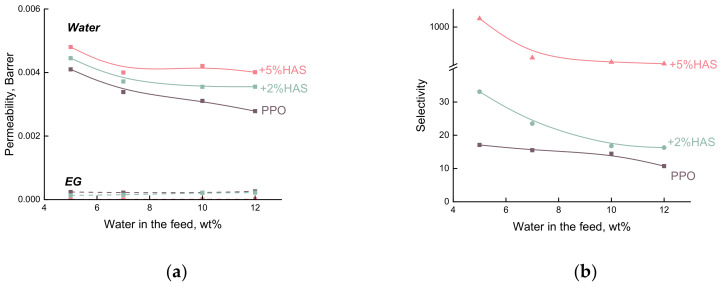
Dependence of (**a**) water and EG permeability and (**b**) selectivity (*α_water/EG_*) on water concentration in the feed for the pervaporation of EG/water mixture using the PPO-based membranes, 50 °C.

Figure 6 shows driving force normalized properties of the PPO-based membranes as the dependencies of water and EG permeability and membrane selectivity (αwater/EG) on water concentration in the feed. The permeability of the hybrid membranes depends on the concentration of water in the feed, even when the driving force contribution is removed. The level of water permeability is much higher than EG permeability. Furthermore, permeability decreases with increasing water concentration in the feed, in contrast with the course of the total flux (Figure 5a). Water permeability increases rapidly with HAS inclusion, as shown in Figure 6a. These results could be due to the significant difference between the sizes of water molecules and EG molecules. This should lead to improved membrane selectivity. Figure 6b shows that the selectivity (αwater/EG) increases with the growth of HAS content up to 5 wt%.

**Figure 7:**
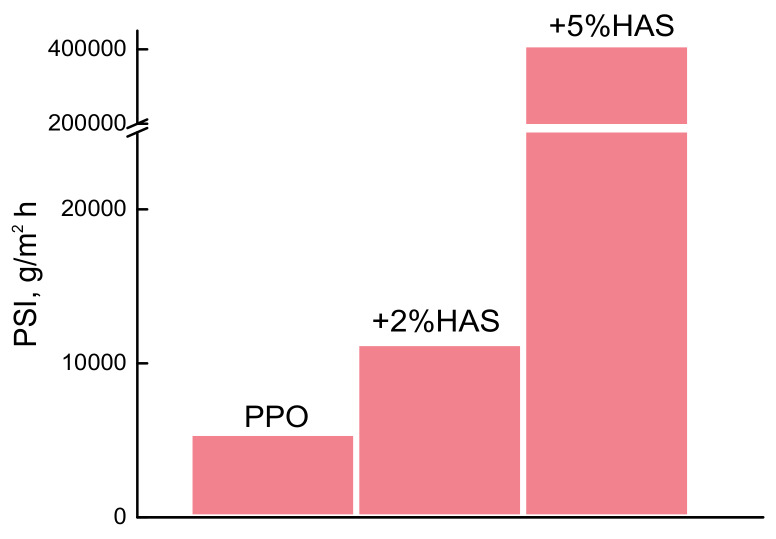
Pervaporation separation index of PPO-based membranes in pervaporation of EG/water mixture containing 5 wt% water, 50 °C.

The membrane efficiency was evaluated using the pervaporation separation index (PSI) which includes magnitudes of both total flux and separation factor. Figure 7 shows data on PSI for three membranes PPO, PPO/HAS (2%), and PPO/HAS (5%) in pervaporation of EG/water mixture containing 5 wt% water. The optimal transport properties were found for PPO/HAS membrane containing 5 wt% HAS.

The transport properties of the PPO/HAS (5%) membrane was compared with literature data for pervaporation separation of the EG/water mixture (10 wt% water). Table 4 lists data on PSI, total flux, and separation factor that had been obtained for different polymer membranes [7,8,9,36,37,38,39,40,41]. The PPO/HAS (5%) membrane shows higher separation efficiency in the EG dehydration as compared with most published data, but it has moderate flux. Although several membranes show higher fluxes, their separation factors are much lower as compared with the PPO/HAS membrane. The presented results indicate the promising application of PPO/HAS (5%) for the EG dehydration. To improve the performance of the membrane for industrial application, it would be necessary to produce composite or hollow fiber membranes with a thin selective layer. Composite bilayer membranes can be prepared by casting a PPO/HAS solution in chloroform onto the surface of a porous substrate, which can be an ultrafiltration polysulfonamide membrane (UPM) produced by Vladipor, Russia, similar to [43,44].

## 4. Conclusions

In the present work, hybrid membranes based on PPO modified with heteroarm stars (HAS) were prepared and studied. The HAS consist of a small branching center fullerene C_60_ and twelve arms of different nature, six arms of non-polar PS and six arms of PTBMA. The morphology, physical and transport properties of membranes in the pervaporation of water/EG mixtures were examined. It was shown that the inclusion of star macromolecules into the PPO leads to a change in the membrane structure due to a disturbance of the initial packing of polymer chains. As such, it can contribute to the formation of a unique transport channel system. Inclusion of HAS macromolecules containing polar arms (PTBMA) leads to the formation of supramolecular clusters due to the intra- and intermolecular segregation of polar chains, which in turn create zones in the membranes that provide selective migration of separated molecules.

The transport properties were studied in the pervaporation of EG/water mixtures containing up to 12 wt% water in the feed; all PPO/HAS membranes produce permeate enriched with water. The growth of HAS content up to 5 wt% in the PPO matrix leads to an increase in the total flux and a prominent increase in the separation factor. The PPO/HAS (5%) membrane exhibits strongly selective properties in the EG dehydration as compared with reported data. The high-water selectivity of the modified membranes is ascribed to the fact that the EG sorption changes the internal structure of the walls in the transport channels. Moreover, water diffusion is facilitated due to the hydrogen bonding with the hydroxyl groups of EG. The data on the transport properties of PPO membranes modified with small amounts of heteroarm star macromolecules give grounds to classify HAS as promising fillers for diffusion membranes.

## Figures and Tables

**Table 1:** Physical parameters of membranes.

Membrane	Density, g cm^−3^	Water Contact Angle, °	EG Contact Angle, °	Critical Surface Tension, mJ/m^2^		
				*σ^p^_s_*	*σ^d^_s_*	*σ_s_*
PPO	1.057	89	63	3.9	24.0	27.9
PPO/HAS (2%)	1.060	91	65	3.2	24.1	27.3
PPO/HAS (5%)	1.064	93	68	2.9	22.6	25.5

**Table 2:** Physicochemical properties of liquids.

Liquid	Mol. Weight	Density, g/cm^3^	Mol. Volume, cm^3^/mol	Dynamic Viscosity, mPa·s	Solubility Parameter *δ*, (J/cm^3^)^1/2^
Water	18.0	0.997	18.0	1.0	49.6
Ethylene glycol	62.1	1.114	55.6	26.0	32.9

**Table 3:** Equilibrium sorption degree of liquids by membranes.

Membrane	Equilibrium Sorption Degree, g Liquid/100 g Polymer	
	Water	Ethylene Glycol
PPO	0	4.2
PPO/HAS (2%)	1.2	4.9
PPO/HAS (5%)	2.9	5.4

**Table 4:** Comparison of transport properties of polymer membranes in the pervaporation of the EG/water mixture (water content 10 wt%).

Membrane	T, °C	PSI, kg/m^2^ h	Separation Factor *β_water/EG_*	Flux, g/m^2^ h	Ref.
CS/PS	35	31	104	300	[10]
PBI/PEI	60	203	1763	115	[11]
PVA	30	208	802	26	[12]
BP-SILM-70	30	103	1014	102	[12]
SO3H-MIL-101-Cr@PD-PVA	70	1566	2900	540	[17]
PA-polyelectrolytes	22	5	415	12	[45]
PVA/PS	60	355	987	360	[46]
PEC NPM	60	681	470	1453	[47]
GFT1001(PVA/PAN)	75	249	1116	224	[48]
DEG167(PVA/PAN)	75	495	991	500	[48]
PIM-1	30	17	92	186	[49]
PPO/HAS (5 wt%)	50	231	11,240	20.6	This work

CS, chitosan; PS, polysulfone; PA, polyamide; PVA, poly(vinyl alcohol); SO3H-MIL-101-Cr, a MOF with SO_3_H groups; BP-SILM-70, buckypaper supported ionic liquid membrane; PEC NPM, polyelectrolyte complex nanoparticles; GFT1001 and DEG167, commercial composite membranes with an active layer of crosslinked PVA and a substrate of polyacrylonitrile microporous membrane; PIM-1, polymer of intrinsic microporosity; PBI, polybenzimidazole; PEI, polyethyleneimine.
